# High abundances of neurotrophin 3 in atopic dermatitis mast cell

**DOI:** 10.1186/1745-6673-4-8

**Published:** 2009-04-22

**Authors:** David Quarcoo, Tanja C Fischer, Nora Peckenschneider, David A Groneberg, Pia Welker

**Affiliations:** 1 Institute of Occupational Medicine, Charité – Universitätsmedizin Berlin, Free University and Humboldt University, D-14195 Berlin, Germany; 2 Department of Dermatology and Allergy, Charité – Universitätsmedizin Berlin, Free University and Humboldt University, D-10115 Berlin, Germany; 3 Institute of Anatomy, Charité – Universitätsmedizin Berlin, Free University and Humboldt University, D-10115 Berlin, Germany

## Abstract

**Background:**

Neurotrophin 3 (NT-3) is a member of the neurotrophin family, a group of related proteins that are known to regulate neuro-immune interactions in allergic diseases. Their cellular sources and role in the recruitment of mast cell precursors in atopic dermatitis have not been characterized in detail so far.

**Objective:**

Characterize NT-3 on a transcriptional and translational level in individuals with atopic dermatitis with special focus on mast cells.

**Methods:**

To meet this objective NT-3 levels in the serum of AD patients were measured, the effect of NT-3 on keratinocytes was evaluated and the gene expression and regulation assessed using ELISA, immunohistochemistry and RNA quantification.

**Results:**

Systemic levels of NT-3 were found to be higher in individuals with AD as compared to healthy controls. A distinct genetic expression was found in the various cells of the skin. In lesional mast cells of individuals with atopic dermatitis an increased amount of NT-3 was apparent. Functional *in vitro *experiments demonstrated that NT-3 stimulation led to a suppression of IL-8 secretion by HaCat cells.

**Conclusion:**

These findings could imply a role for NT-3 in the pathogenesis of allergic skin diseases.

## Introduction

The atopic dermatitis (AD) is a persistent relapsing inflammatory skin disease associated with dry skin, itching and an ever increasing prevalence, particularly in the age group of early childhood [1]. AD has been grouped into an intrinsic and extrinsic type according to the presence of IgE-mediated sensitization which is found in the extrinsic type. Accumulating Data have suggested that the nervous system influences the course of AD through emotional stress, altered patterns of skin innervation, and abnormal expression of neuromediators [2,3]. Neurotrophins, a family of structurally and functionally related polypeptides, act as mediators in the interactions between both immune and nerve cells [4]. The effect of neurotrophins is mediated by two types of receptors that vary in terms of ligand binding specificity. While the low affinity neurotrophin receptor P75 is capable of binding to all neurotrophins with equivalent affinity, tyrosine kinase (Trk) family members exhibit ligand selectivity. The TrkC receptor appears be unique in binding only one type of neurotrophin and none of the other related ligands [5]. The bound ligand, neurotrophin (NT)-3 is a 119 amino acid basic protein and has about 50% homology to the nerve growth factor (NGF) as well as to the brain-derived neurotrophic factor (BDNF) and NT-4, three other members of this family [6]. NT-3 binds to TrkC as its high affinity tyrosine kinase receptor and shows low affinity interactions with the low affinity NT receptor P75 and TrkA and TrkB, the high affinity receptors for NGF and BDNF/NT-4, respectively [7]. From cells that can be found in the skin, fibroblasts and human epidermal keratinocytes produce NT-3 *in vitro *[8]. Also, NT-3 acts as a growth factor for human melanocytes *in vitro *[9].

Bone marrow-derived, tissue resident mast cells have been shown to increase in numbers in a wide variety of inflammatory and neoplastic conditions. They play a central role in the pathogenesis of AD [10]. It has been demonstrated that the interaction between mast cells and nerves in patients with AD is mediated by neuropeptides like substance P, calcitonin gene related peptide or vasoactive intestinal peptide [2-4]. In addition, there is recent evidence that besides these short peptides also NTs are potentially mediators of nerve-mast cell interaction. Skin mast cells were described to release NGF [11,12] and the human mast cell line (HMC-1) produces besides NGF also BDNF and NT-3 [13]. In the same article it was also shown, that HMC-1 cells express the NT receptors TrkA, TrkB and TrkC [13]. Therefore, mast cells are not only a source, but also possible effector for NTs. Up to the present, there are only rare information which other kinds of cutaneous cells are able to produce NTs [14,15]. NGF is expressed by several cell types such as keratinocytes, fibroblasts and melanocytes [16]. One study demonstrated the up regulation of NT-4 expression in the keratinocytes of skin from patients with AD, whereas NT-3, expressed in dermal fibroblasts, remained unchanged [17].

Here we investigate which skin cell types have the capacity to produce NT-3 to obtain more information about the network of NTs as a part of the cytokine network in the skin. Modified expression in the skin of patients with AD compared to normal skin give new insides in the role of NT in the pathogenesis of this disease.

## Methods

### Tissue

Biopsies from 45 patients with atopic dermatitis (>16 years, mean age 38.5 years, 24 females, 21 males) and 23 normal controls (>16 years, mean age 42.8 years, 13 females, 10 males) were examined. Atopic dermatitis diagnosis was based on the criteria of Hanifin [18], and routinely performed histopathological examination revealed characteristic inflamed eczematous lesions. The SCORAD of the atopic dermatitis patients was >25 (moderate or severe). Cutaneous keratinocytes, endothelial cells, fibroblasts, melanocytes, and MC were obtained from human foreskin or breast skin of non-atopic patients undergoing cosmetic surgery and isolated as described previously [19]. The skin MCs were enriched (95% purity) using immunobeads (Dynal, Hamburg, Germany) coated with a c-Kit antibody YB5.B8 and magnetic cell sorting [20]. The human HaCaT keratinocytes cell line was kindly provided by N. Fuseing (Heidelberg, Germany) [21]. All studies were performed according to the declaration of Helsinki, after patients had given their informed consent.

### RNA-Isolation

1 × 10^6 ^cells were lysed and total RNA was prepared using the RNeasy-total-RNA-kit (Qiagen, Hilden, Germany). After digestion of genomic DNA by treatment with DNAase, cDNA was synthesized by reverse transcription of 5 μg total RNA, using a cDNA synthesis kit (InVitrogen, Stade, USA).

### Reverse-Transcription Polymerase Chain Reaction

The following sets of oligonucleotide primers were used to amplify cDNA (expected fragment lengths are given in parenthesis): **GAPDH**: 5' GAT GAC ATC AAG AAG GTG GTG and 5'GCT GTA GCC AAA TTC GTT GTC (197 bp) [19]; **NT-3**: 5'CCGCCCTTGTATCTCATGGA, and 5'CCGTGATGTTCTGTTCTGTCGCC (354 bp) [22]. Amplification was performed using Taq polymerase (GIBCO) over 24–38 cycles with an automated thermal cycler (Perkin Elmer, FRG). Each cycle consisted of the following steps: denaturation at 94°C, annealing at 58°C (GAPDH), 63°C (NT-3) and extension at 72°C for 1 min each. PCR products were analyzed by agarose gel electrophoresis and enzymatic digestion, using standard techniques.

### Immunocytochemical staining

Anti-NT-3 monoclonal antibodies were used (1:100, rabbit, sc-547 from Santa Cruz Biotechnology, Santa Cruz, USA) to perform immunohistochemistry using the APAAP technique. To quantify mast cell numbers and assess mast cell p75 staining intensity, previously described and validated protocols were used [23]. In brief, sections were evaluated by two independent investigators. Counting of nucleated stained cells was performed using a raster covering 1/16 mm^2 ^at 1: 400 magnifications in at least five microscopic fields. Counts were expressed as stained cells per mm^2^. Quantification of mast cell staining intensity for neurotrophins and their receptors was performed using an intensity ranging from 0 to 3.5, as previously described and validated [24-26]. Measurement of intensity was performed for at least four slides of each patient and control subject in a blinded fashion.

### Cell culture and stimulation

Human HaCaT keratinocyte cells were kept in Dulbeccos Eagle's medium (Gibco, Karlsruhe, Germany), supplemented with 5% fetal bovine serum (Biochrom, Berlin, Germany), 4 mM glutamine, and 100 U penicillin and streptomycin per mL (both from Gibco, Karlsruhe, Germany) [21]. Cells were seeded at 2 × 10^6 ^cells/cm^2 ^in culture plates, and the medium was routinely changed every 3 or 4 days. As described, in some experiments, the medium was removed after 3 day of culture and, following another 24-h culture with different concentrations of NT-3, the supernatants were collected to compare the IL-8 quantities.

### Cytokine measurements

Serum NT-3 levels in serum of individuals with AD (n = 10) were compared with healthy controls (n = 12) using a commercially available ELISA kit from R&D Systems (Minneapolis, USA). Cell supernatants were analyzed for IL-8 contents with a commercially available enzyme-linked immunoabsorbent assay, ELISA Kit (Quantikine, R&D systems, Bad Nauheim, Germany). Values of duplicate measurements fluctuated within a very narrow margin (< 5%). The results were adjusted to viable cell counts and expressed as means ± SD of four different experiments.

### Statistics

Results of the different parameters and groups are expressed as mean ± s. e. m unless stated differently. Statistical significance was calculated using the unpaired two-tailed t-test.

## Results

### NT-3 serum levels in individuals with AD

**Figure 1:**
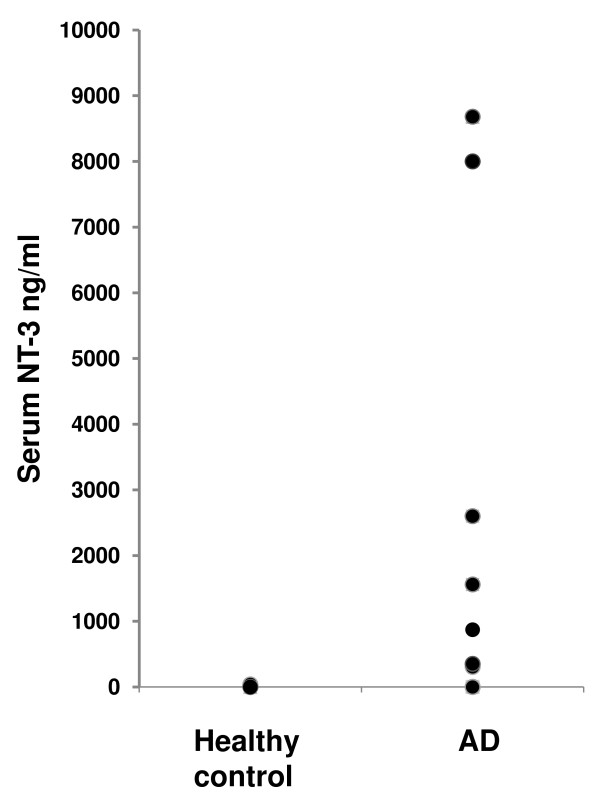
**NT-3 protein plasma levels**. Plasma levels of NT-3 [in ng/ml] in healthy individuals (*n *= 12) compared to patients with AD (*n *= 10) detected by ELISA.

Serum NT-3 levels in serum of individuals with AD were compared with healthy controls using a commercially available ELISA kit. As depicted in Fig. 1 significantly higher amounts of NT-3 were found in the serum of the AD group as compared to the control group.

### NT-3 mRNA expression in cutaneous cells

**Figure 2:**
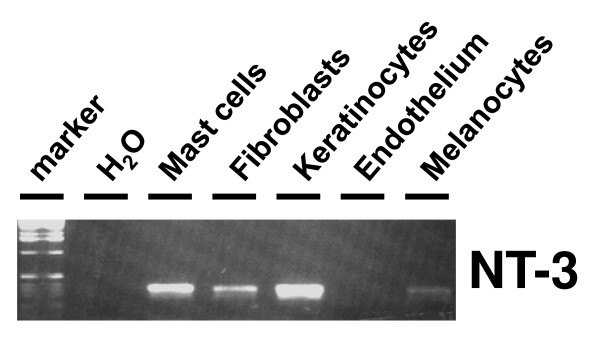
**NT-3 mRNA expression in isolated cutaneous cells**. Expression of mRNA specific for NT-3 (RT-PCR) in isolated skin keratinocytes, fibroblasts, endothelial cells, melanocytes, and mast cells (representative results from four different experiments).

To assess the cellular expression of NT-3 mRNA in human skin of non-atopic patients, different cell populations were freshly isolated and subjected to RT-PCR. Using NT-3 specific primer pairs, repeated (n = 4) RT-PCR experiments were performed and NT-3 specific amplification products with a length of 354 bp were detected in different cell types. Strong NT-3 specific mRNA signals were present in mast cells, keratinocytes, and fibroblasts, weaker signals were found in melanocytes, whereas in endothelial cells no signal was detected (Fig. 2). The PCR product identities were confirmed by direct sequencing, which revealed identity with the published sequences (Data not shown).

### Mast cell-specific of NT-3 expression in normal and lesional topic dermatitis skin

**Figure 3:**
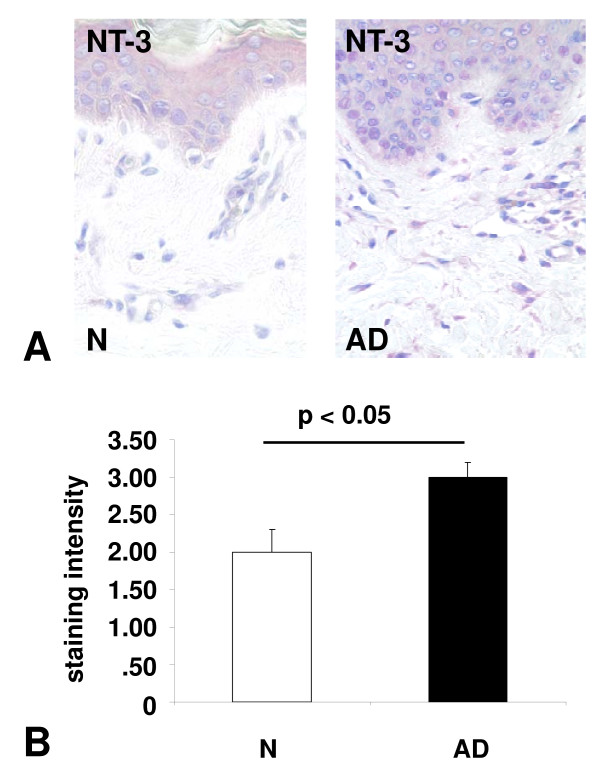
**NT-3 expression in atopic dermatitis lesional mast cells: (a) Immunohistochemical staining (APAAP) of normal skin sections (N) and from patients with atopic dermatitis (AD) using specific antibodies against NT-3**. (40 × magnification). (b) Quantitative analysis of NT-3 immunohistochemical staining (APAAP) of normal skin sections and from patients with atopic dermatitis. Black bars, atopic dermatitis; open bars, controls.

To assess the protein expression of NT-3 in human skin in situ, NT-3 immunohistochemistry was performed, leading to the identification of NT-3 in MCs of both normal and atopic dermatitis MCs. The transcriptional regulation of NT-3 in lesional MCs of atopic dermatitis patients was assessed using quantitative immunohistochemistry, and significant changes in protein expression were found between normal and atopic dermatitis MCs. Quantitative immunohistochemistry demonstrated significant changes in protein expression from 2,0 ± 0.3 (control) to 3.0 ± 0.2 (AD) for NT-3 (p < 0.05) (Fig. 3).

### Incubation of HaCaT cells with increasing concentrations of NT-3

**Figure 4:**
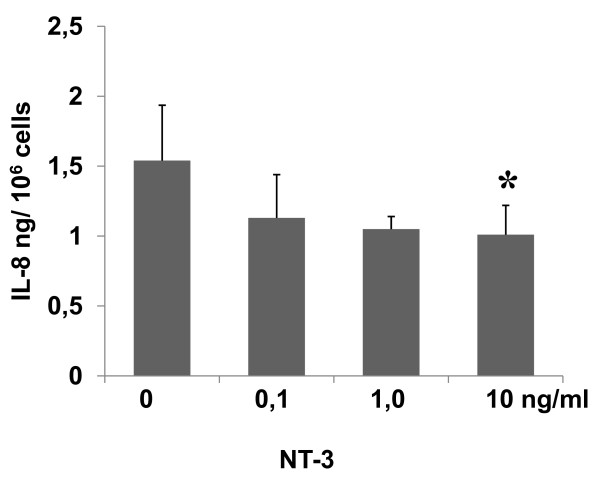
**Functional effects of increasing NT-3 levels on HaCaT keratinocytes secretion**. Release of IL-8 [in ng/10^6 ^cells] after stimulation with different levels of NT-3.

To assess the functional effects of a decreased NT-3 secretion, *in vitro *studies were performed that assessed the secretory activity of HaCaT keratinocytes, and significant differences were found. The secretion of IL-8 in HaCaT cells after stimulation with different levels of NT-3 decreased in a dose-dependent manner. The reduced NT-3 stimulation from 10, 1 and 0,1 ng/mL led to an increase in IL-8 secretion that was significant (P < 0.05) (Fig. 4).

## Discussion

In the present study, we have provided evidence that in atopic dermatitis, NT-3 is up regulated on a systemic level as well as on a tissue level, suggesting that there is an increased release of NT-3 in allergic disease of the skin.

Since it's cloning in 1990 by Jones and Reichard the neurotrophin-3 has been for the most part subject of neuroscience research [27]. Here, complex regulation patterns were found in regard to outgrow and maintenance of neurons [28,29]. In the ontogenesis a lack of neurotrophin support leads to specific deficits in sensory neuron number and peripheral innervation patterns [28,30,31]. Mice that are deficit in NT3 lack up to 70% of their sensory neurons during embryonic development and certain types of sensory complexes [28].

In the adult organism elevated systemic NT-3 levels have been linked to various diseases. Thus, Walz et. al. show that NT-3 was elevated in patients with psychiatric illnesses [32]. In a study investigating the systemic NT-3 level in asthmatic patients the authors not only found an association between higher levels of NT-3 and the disease, but also demonstrated a significant drop in the elevated serum level of the neurotrophin after treatment of the underlying asthma [33]. In line with the last study in the current study elevated serum levels of NT-3 were detected in the AD patients. It has been shown that inflammatory processes induce NT-3 [34], since in AD this takes place in the skin it represents a potential site for the systemic NT-3 release.

The roles of NT-3 in the biology of the skin have not been fully elucidated yet. The best-established role for NT-3 is the support and maintenance of sensory nerve endings and accessory organs [35,36]. In the current study NT-3 expression in different cell populations of the skin was demonstrated. Although, other studies that have detected expression of NT-3 in cell cultures of mast cells and fibroblasts, a publication by Innominnato et. al. found that in humans melanocytes expression of NT-3 only took place after the malign transformation of the cells [37].

In the current study endothelial cells were the only cell type that did not express NT-3. The expression of neurotrophins and their receptors was investigated in large arteries of the lung and other sites in a study by Ricci and coworker. It was shown that the expression of NT-3 was mainly located in the tunica media and tunica adventitia where it was hypothesis to take part in the regulation of vascular mobility, owing to the fact that TrkC and P75 were abundantly expressed in vascular smooth muscle [38]. Lacking the large amount of smooth muscle it might be assumed that in the skin the expression is low in the fine dermal vessels.

A study that measured the quantity of neurotrophins in different tissues throughout life of mice found that NT-3 is not only expressed in various tissues after birth, but also retains detectable values throughout the lifetime of the organism. In particular in the thymus, a site inhabited by cells of the immune system the expression of NT-3 remains high [39]. An emerging body of evidence suggests that NT-3 is able to interfere in immunological processes. In this line Barouch and coworker established that NT-3 was not only spontaneously expressed by leukocytes, but was further increased after the stimulation of the cells with LPS [34]. A phenomenon found to be also true for other neurotrophins [40]. The direction of inflammation played an important role in a study by Besser et. al where he showed that only human Th2 clones can be stimulated to release NT-3 by the cytokine IL-4, the paramount Th2 cytokine [41]. Therefore, the release of NT-3 might affect the activity and direction of an inflammatory process. Also, NT-3 might enhance survival and triggers the local immune cells in the skin, a capacity which has been shown for eosinophiles and monocytes [42]. Also other cell types, both naive and stimulated have been shown to express NT-3 [9,43-45]. Mast cells that have been proposed to play an important role in the interface between the immune and the nervous system are able to express NT-3 [13]. Metz and coworker demonstrated that more mast cells can be found in the skin of transgenic mice over expressing NT-3 [46]. Conversely TrkC knockout mice present only with a slight reduction in mast cell numbers, which suggested that NT-3 and its high affinity Trk receptor play a part in up regulating the number of MCs, but do not control numbers of basal MCs.

In the current study we found evidence for a functional role for NT-3 in AD. Interestingly, NT-3 stimulation led to a decreased secretion of IL-8 in HaCaT cells. In a recent study Nomura et. al. has attributed the down regulation of pro-inflammatory cytokines like IL-8 to the increased susceptibility of the AD skin to microorganisms, and suggested a new fundamental rule that may explain the mechanism for frequent infection in other Th2 cytokine-mediated diseases [47]. Because previous studies demonstrated a close interaction between MCs and keratinocytes in atopic dermatitis [48], it can be assumed that the presently observed increase in NT-3 production in MCs may also have functional effect on keratinocytes IL-8 secretion in states of atopic dermatitis. Enhanced NT-3 levels may lead to decreased IL-8 production by keratinocytes as shown by the HaCaT *in vitro *studies and thus to functional consequences in AD.

Taken together, the present study presents data on the expression and function of NT-3 in lesional cutaneous mast cells in AD, thus providing data proposing possible regulatory mechanisms involved. Mast cell-nerve interactions may thus contribute crucially to the development and progression of the chronic inflammatory lesions in atopic dermatitis.

## Abbreviations

AD: Atopic Dermatitis; NT: neurotrophin; MC: mast cell; Trk: tyrosine kinase; RT-PCR: reverse transcription polymerase chain reaction; NGF: nerve growth factor; BDNF: brain derived neurotrophic factor; SCORAD: SCORing Atopic Dermatitis

## Competing interests

The authors declare that they have no competing interests.

## Authors' contributions

DQ conceived of the study, participated in the design and co-ordination of the study, interpreted the data and drafted and prepared the manuscript. TCF and PW helped to conceived of the study. PW, NP performed the analysis. DAG and PW helped to interpret the data. All authors read and approved the final manuscript.
